# The combination of cigarette smoke and solar rays causes effects similar to skin aging in a bilayer skin model

**DOI:** 10.1038/s41598-023-44868-z

**Published:** 2023-10-20

**Authors:** Alexe Grenier, Mathieu C. Morissette, Patrick J. Rochette, Roxane Pouliot

**Affiliations:** 1 Centre de Recherche en Organogénèse Expérimentale de l’Université Laval/LOEX, Axe Médecine Régénératrice, Centre de Recherche du CHU de Québec-Université Laval, Quebec City, QC G1J 1Z4 Canada; 2 Faculté de Pharmacie, Université Laval, Quebec City, QC G1V 0A6 Canada; 3 Québec Heart and Lung Institute-Université Laval, Quebec City, QC G1V 4G5 Canada; 4 Département de Médecine, Faculté de Médecine, Université Laval, Quebec City, QC G1V 0A6 Canada; 5 Département d’Ophtalmologie et ORL-Chirurgie Cervico-Faciale, Faculté de Médecine, Université Laval, Quebec City, QC G1V 0A6 Canada

**Keywords:** Cell biology, Biochemistry, Biomedical engineering

## Abstract

Skin aging is a multifactorial process influenced by internal and external factors. The contribution of different environmental factors has been well established individually in the last few years. On the one hand, man is rarely exposed to a single factor, and on the other hand, there is very little knowledge about how these extrinsic factors may interact with each other or even how the skin may react to chronic exposure. This study aimed to evaluate the effect on skin aging of a chronic co-exposure of tissue-engineered skin substitutes to cigarette smoke extract (CSE) and solar simulator light (SSL). Skin substitutes were reconstructed according to the self-assembly method and then exposed to CSE followed by irradiation with SSL simultaneously transmitting UVA1, visible light and infrared. When skin substitutes were chronically exposed to CSE and SSL, a significant decrease in procollagen I synthesis and the inhibition of Smad2 phosphorylation of the TGF-β signaling pathway were observed. A 6.7-fold increase in MMP-1 activity was also observed when CSE was combined with SSL, resulting in a decrease in collagen III and collagen IV protein expression. The secretory profile resulting from the toxic synergy was investigated and several alterations were observed, notably an increase in the quantities of pro-inflammatory cytokines. The results also revealed the activation of the ERK1/2 (3.4-fold) and JNK (3.3-fold) pathways. Taken together, the results showed that a synergy between the two environmental factors could provoke premature skin aging.

## Introduction

Skin aging is a complex phenomenon involving several mechanisms and caused by both internal and external factors, defined as intrinsic and extrinsic aging, respectively^1^. The latter regroups various extrinsic factors that are dangerous for human health. The exposition to these factors can be define as the skin aging exposome. The exposome of skin aging began to gain more interest in 2016–2017 when Dr. Jean Krutmann and his colleagues defined this concept as all the external and internal factors, as well as their interactions, that affect an individual during his life and the body’s response to these factors that lead to the biological and clinical signs of skin aging^1^. Extrinsic factors such as solar rays, tobacco smoke, atmospheric pollution, and other factors less studied but potentially harmful, such as nutrition, stress, lack of sleep and temperature, can contribute to the skin aging exposome.

It has been known for decades that exposure to ultraviolet (UV) radiation, representing 5% of the solar spectrum, leads to clinical hallmarks of skin aging, proving their undeniable role in this process. Both UVB (280–315 nm) and UVA (315–400 nm) contribute to extrinsic skin aging to different extents. UVB, representing 5% of UV radiation reaching the earth surface, is the most energetic radiation reaching the skin, but it penetrates only the epidermis and the papillary dermis. On the other hand, UVA, even if less energetic, represents 95% of the UV radiation reaching the skin^1,2^. Long-wave UVA, namely UVA1 (340–400 nm), can penetrate deeper into the skin and have a direct effect on dermal components ^3^. Other wavelengths present in sunlight also contribute to skin aging. Indeed, infrared radiation (IR; > 800 nm), which penetrates beyond the dermis, contributes to skin aging by inducing the production of matrix metalloproteinases (MMPs), decreasing the antioxidant content of the skin, and modulating signaling pathways such as extracellular signal-regulated kinases 1/2 (ERK1/2), p38 mitogen-activated protein kinases (MAPKs), c-Jun N-terminal kinases (JNKs), phosphoinositide 3-kinases/AKT (PI3K/AKT), signal transducer and activator of transcription 3 (STAT3) and interleukin (IL)-6^4,5^. In addition to IR, visible light (VL; 400–700 nm) can also play a critical role in premature skin aging. It has been shown that VL, more precisely the blue light spectrum (400–500 nm), can provoke oxidative stress via the production of reactive oxygen species (ROS) and induce MMP production^6,7^.

Cigarette smoke is another environmental factor well known for causing premature skin aging, as showed by the accentuated appearance of wrinkles in smokers^8,9^. Cigarette smoke accelerates skin aging by provoking oxidative stress, impairing the growth and proliferation of fibroblasts, inducing MMP production, and thereby decreasing the extracellular matrix content^10,11^.

Despite the various studies that have shown the contribution of various individual environmental factors to skin aging, humans are rarely exposed only to one factor, and there is very little knowledge about the interaction, or even synergy, between the different extrinsic factors. A study that investigated long-term exposure (10 months) to cigarette smoke and UV rays in mice reported a disruption of the skin barrier with transepidermal water loss, erythema, and an increased incidence of epitheliomas and squamous cell carcinomas following exposure^12^. This study comes to support that more evidence and a better understanding are needed regarding the interaction between the exposome factors. Research has therefore reached a turning point where it becomes imperative to investigate the various possible interactions in order to better understand the phenomenon of skin aging to which a given individual is subjected. Epidemiological studies have also highlighted a possible interaction between UV exposure and cigarette smoking regarding wrinkle formation^13^. A previous study led by our group showed a significant synergy between cigarette smoke and solar rays in their effect on human keratinocytes, especially on cellular viability and oxidative stress via the production of type II ROS^14^. This led us to believe there would be a harmful effect on the skin, in particular the premature aging of the skin. The aim of this study was to evaluate the impact of this photo-pollution stress on tissue-engineered skin substitutes reconstructed according to the self-assembly method. The effect of the combination of solar radiation and a cigarette smoke extract (CSE) on the protein expression of different skin aging markers was therefore investigated. The mechanisms by which those markers were altered have also been examined in order to have new insight into the interaction between the two factors. To our knowledge, this is the first study in a reconstructed bilayer skin model investigating the consequences of a chronic exposure (once a day during seven days) to both solar rays and cigarette smoke.

## Materials and methods

### Biopsies and cell extraction

The study was approved by the Research Ethics Committee of the “Centre Hospitalier Universitaire (CHU) de Québec-Université Laval” (ethics code: DR-002-1121, protocol renewal approved on December 12th, 2022). The study was also performed in agreement with the Helsinki declaration and informed consent was obtained from all the participants.

Biopsies were obtained during breast reduction surgeries and donors were Caucasian females aged 38, 42, 46, 49 and 52 years old. Epidermis was firstly detached from the dermis of the biopsies after being incubated in thermolysin at 4°C for 16 h. Then, cells were extracted from the biopsies with the isolation method previously described, using thermolysin and then trypsin digestion for keratinocytes and collagenase digestion for fibroblasts^15,16^.

### Cell culture

Primary fibroblasts (passage 4) and keratinocytes (passage 1) were cultured in specific media as previously described^17^. Fibroblasts were first thawed and then seeded at 4 × 10^3^ cells/cm^2^ in 75 cm^2^ cell culture flasks, while keratinocytes were thawed and seeded at 4 × 10^3^ cells/cm^2^ in 75 cm^2^ cell culture flasks on a feeder layer of gamma-irradiated human dermal fibroblasts. Cell cultures were incubated at 37°C in an 8% carbon dioxide (CO_2_) atmosphere. Cell culture media were changed three times per week.

### Skin substitute production

**Figure 1:**
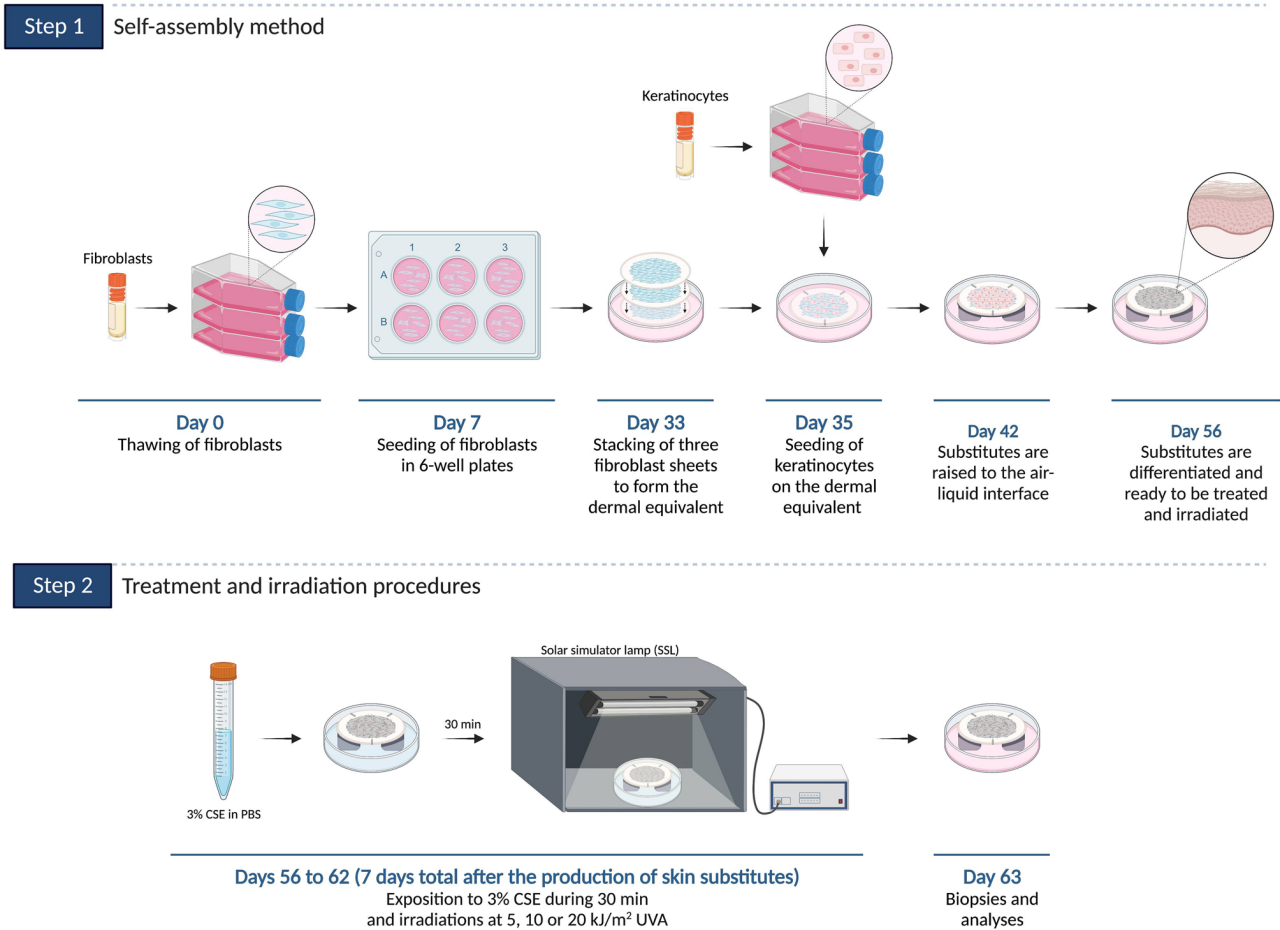
Self-assembly method (step 1) and treatment and SSL irradiation procedures (step 2).

Skin substitutes were produced according to the self-assembly method, partially modified using 6-well plates (Fig. 1, step 1)^17–20^. Fibroblasts at passage 5 from healthy donors were seeded at 1.5 × 10^5^ cells/well and cultured for 26 days in Dulbecco’s Modified Eagle’s Medium (DMEM) supplemented with 10% FB Essence serum (FBe; Seradigm, Salt Lake City, UT, USA), 100 IU/mL penicillin G (Sigma, Oakville, ON, Canada), 25 µg/mL gentamicin (Gemini, West Sacramento, CA, USA) and 50 μg/mL (+)-sodium L-ascorbate (Sigma, St. Louis, MO, USA) until they formed manipulable sheets^17^. Then, three fibroblast sheets were detached and superimposed to form the dermal equivalent. Dermal equivalents were incubated at 37°C with 8% CO_2_ for two more days to allow sheet fusion and thus form the new dermal layer. After this period, keratinocytes at passage 2 from healthy donors were seeded on the dermal equivalent at 1.2 × 10^6^ cells/equivalent to form the epidermal layer and cultured for seven days in a combination of DMEM with Ham’s F12 in a proportion of 3:1 (DMEMH) supplemented with 5% Fetal Clone II serum (Hyclone, Scarborough, ON, Canada), 5 µg/mL insulin (Sigma, St. Louis, MO, USA), 0.4 µg/mL hydrocortisone (Galenova, Saint-Hyacinthe, QC, Canada), 0.212 µg/mL isoproterenol hydrochloride (Sandoz Canada, Boucherville, QC, Canada), 10 ng/mL human epidermal growth factor (EGF; Austral Biological, San Ramon, CA, USA), 100 IU/mL penicillin G (Sigma, Oakville, ON, Canada), 25 µg/mL gentamicin (Gemini, West Sacramento, CA, USA) and 50 μg/mL (+)-sodium L-ascorbate (Sigma) to allow keratinocyte proliferation^17^. Skin substitutes were then raised to the air–liquid interface to promote cell differentiation and obtain the different epidermal layers. At the air–liquid interface, skin substitutes were cultured with medium lacking EGF to obtain a stratified epithelium representative of in vivo skin. Figure 1 (step 1) presents the method.

### Cigarette smoke extract (CSE) preparation

**Figure 2:**
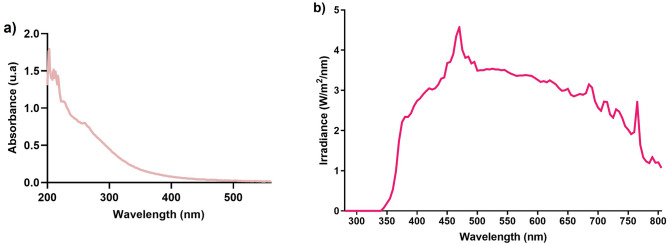
(**a**) UV–visible spectrum of the CSE stock solution and (**b**) irradiance spectrum of the solar simulator light (SSL) with the CGA-345 long-pass filter.

Fresh CSE was prepared each day as described previously^14^. Briefly, using an AGI-30 impinger (Ace Glass Inc., Vineland, NJ, USA), 3R4F reference cigarettes (Tobacco-Health Research Institute, University of Kentucky, Lexington, KY) connected to a 60 mL syringe by a 3-way stopcock were smoked by suction in 20 mL of 1X phosphate buffered saline (PBS)^14^. The absorbance spectra of CSE stock solutions were acquired each time using a scanning UV–visible spectrophotometer (Varian Cary® 50 Bio UV–visible spectrophotometer), after a baseline correction using 1X PBS. Absorbance spectra were recorded in the 200–560 nm range. Each CSE stock solution was normalized according to the UV–visible spectrum presented in Fig. 2a.

### Treatment and SSL irradiation procedures

Fourteen days after being raised to the air–liquid interface, skin substitutes were treated each day throughout a week (7 days) with a solution of CSE for 30 min, in order to allow the incorporation of CSE compounds, at 37°C with 8% CO_2_ in the dark (Fig. 1, step 2). Based on a previous study led by our group^14^, a concentration of 3% (v/v) CSE was chosen for the treatment of skin substitutes. The 3% CSE was obtained by diluting the CSE stock solution (considered as 100%) with 1X PBS. Immediately after the incubation time, skin substitutes were irradiated with an Oriel 1.6 kW solar simulator lamp, with an ozone-free xenon short arc lamp combined with an air mass 1.5G filter (Newport, CA, USA). A Newport’s Colored Glass Alternative long-pass filter with a 345 ± 5 nm cut-on wavelength (CGA-345; Newport, CA, USA) was used to block UVB and UVA2 wavelengths, meaning that only UVA1, VL and IR were transmitted^14^. The irradiance spectrum of the SSL with the long-pass filter can be found in Fig. 2b. Even if the skin substitutes were exposed to the whole solar spectrum minus UVB and UVA2, the SSL doses were expressed in function of UVA. Skin substitutes were exposed to doses of 5, 10 or 20 kJ/m^2^ of UVA, corresponding approximately to a 5-, 10-, or 20-min exposure to the sun at its zenith in Paris (France) at noon, respectively^21^. The treatment and SSL irradiation procedure was performed each day throughout a week (7 days). Skin substitutes treated in 1X PBS only (without SSL irradiation) were used as controls. Figure 1 (step 2) presents the procedure.

After a total of 63 days of culture (with 7 days of treatment and SSL irradiation, from day 56 to day 62 inclusively), skin substitute biopsies and culture supernatants were taken (the day after the last irradiation) and analyzed by histology, immunofluorescence, dot blot, western blot, ELISA and a cytokine array.

### Histological analyses

Skin substitute biopsies from each condition were fixed in HistoChoice® solution (Amresco, Solon, OH, USA) and embedded in paraffin wax. 5 μm thick sections were then cut and coloured with Masson’s Trichrome stain. Two skin substitutes per donor (with at least three donors; 3 < N < 4) for each condition were analyzed. The thickness of the living epidermis and dermis was measured with ImageJ software (National Institutes of Health (NIH), Bethesda, MD, USA) on the stained sections. For the thickness measurements, at least three different cell populations were analyzed, and for each of them, two representative pictures per condition were taken and 10 measurements per picture were made for a total of 60 measurements per condition.

### Dot blot and western blot analyses

Tissues preparation and protein extraction were performed as described previously^22,23^. The dermis and the epidermis from skin substitutes were separated manually using forceps and scalpels. Tissues were ground into fine powder using cryogenic grinding with a CryoMill MM400 (Retsch, Newtown, PA, USA). Proteins were then extracted from the ground tissues with a lysis buffer (radioimmunoprecipitation assay buffer; RIPA buffer) containing a protease inhibitor cocktail (Roche, Mannheim, Germany), 500 µL for the epidermis and 250 µL for the dermis^22,23^.

The samples for the dot blot analyses were prepared so that 5 μg of protein per sample was loaded in the Bio-Dot Apparatus, on a nitrocellulose membrane. After the preparation of the dot blot membranes and the 1 h blocking in tris-buffered saline (TBS) with 0.05% Tween 20 (TBST) and 5% non-fat milk, dot blot membranes were incubated at room temperature for 1 h with the primary antibody and for another hour with the secondary antibodies. The primary and secondary antibodies used for the dot blot analyses are listed in Supplementary Table S1.

The samples for the western blot analyses were prepared so that 40 μg of protein per sample for epidermis samples and 20 µg of protein per sample for dermis samples was loaded in a 10% reducing SDS-PAGE gel. Proteins were then transferred to an Immun-Blot PVDF membrane (Bio-Rad Laboratories, Mississauga, ON, Canada). After the transfer, membranes were blocked for 1 h in TBST and 5% non-fat milk and then incubated overnight with the primary antibody at 4°C. Membranes were then incubated for 1 h at room temperature with the secondary antibody. The primary and secondary antibodies used for the western blot analyses are listed in Supplementary Table S1.

The detection of dot blot and western blot was performed with ECL Prime Western Blotting Detection Reagent (GE Healthcare, Little Chalfont, UK) and the Fusion Fx7 imager (Montreal Biotech Inc, Québec, Canada). The quantification of the blots was performed by densitometry using ImageJ software (National Institutes of Health (NIH), Bethesda, MD, USA).

### Secretory profile analyses

Cell culture supernatants without serum were collected 24 h after the last SSL irradiation (day 7 of treatment and irradiation) and kept at -80°C until the analysis. The evaluation of procollagen I synthesis, and IL-6 and TIMP-1 production were assessed by ELISA with the Human Pro-Collagen I alpha 1 ELISA kit (#ab210966, Abcam, Waltham, MA, USA), the Human IL-6 ELISA kit (#ab178013, Abcam, Waltham, MA, USA) and the Human TIMP1 ELISA kit (#ab187394, Abcam, Waltham, MA, USA), while MMP-1 activity was evaluated with the Human Active MMP-1 Fluorokine E Kit (#F1M00, R&D Systems, Minneapolis, MN, USA) according to the manufacturer's instructions. Procollagen I, IL-6 and TIMP-1 detection was carried out with a microplate reader (SpectraMax Plus 384 Microplate Reader, Molecular Devices, San José, CA, USA), while MMP-1 detection was performed with a spectrofluorometer (Varioskan Flash, Thermo Electron Corporation, Waltham, MA, USA). A cytokine array was also performed on cell culture supernatants to evaluate the model’s secretome after treatment and SSL irradiation at a broader range. The Human XL Cytokine Array (R&D Systems, Minneapolis, MN, USA) was used according to the manufacturer’s instructions. The four membranes were developed in an autoradiography film cassette using an SRX-101A medical film processor (Konica, Tokyo, Japan). The membranes were exposed to the X-ray films for 2, 5 or 10 min and scanned with an Epson Perfection V750 Pro image scanner (Epson, Markham, ON, Canada). The dot signal intensity was analyzed with Image StudioLite software (LI-COR, Lincoln, NE, USA).

### Statistical analysis

Results are expressed as means ± standard deviation (S.D.). Statistical differences between conditions were analyzed with a one-way Analysis of Variance (ANOVA) followed by Tukey’s post hoc test. Results were considered significant when *p* < 0.05. Statistical analyses were performed with Prism software V5 (GraphPad Prism Software, San Diego, CA, USA).

## Results

### The synergy impairs the extracellular matrix of the dermis

**Figure 3:**
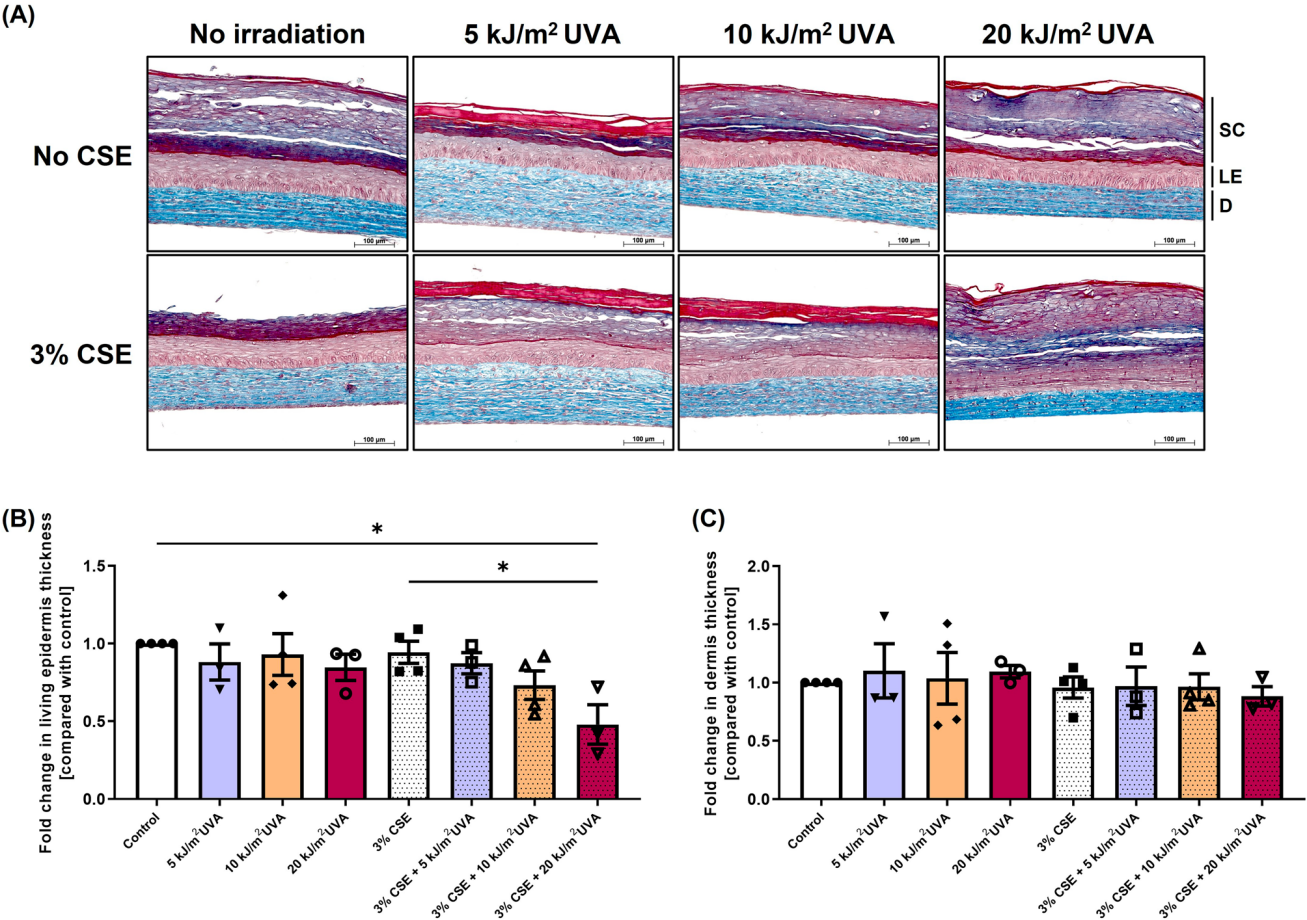
Effect of cigarette smoke extract (CSE) and SSL irradiation on reconstructed skin substitute morphology. (**A**) Histological analyses of Masson’s trichrome-stained skin substitutes. Stratum corneum (SC) in dark blue/purple, living epidermis (LE) in violet/pink and dermis (D) in light blue. Objective 10X, scale bar: 100 μm. (**B**) Fold change in the thickness of the living epidermis and (**C**) of the dermis. Fold change is defined as the ratio of exposed substitutes’ thickness value to the control (without treatment) thickness value. Two substitutes for each condition were analyzed and confirmed with at least three different cell populations (3 ≤ N ≤ 4, 6 ≤ n ≤ 8). Data are presented as means of the different cell populations ± S.D. Statistical significance was determined using one-way ANOVA followed by Tukey’s post hoc test, * *p* value < 0.05.

**Figure 4:**
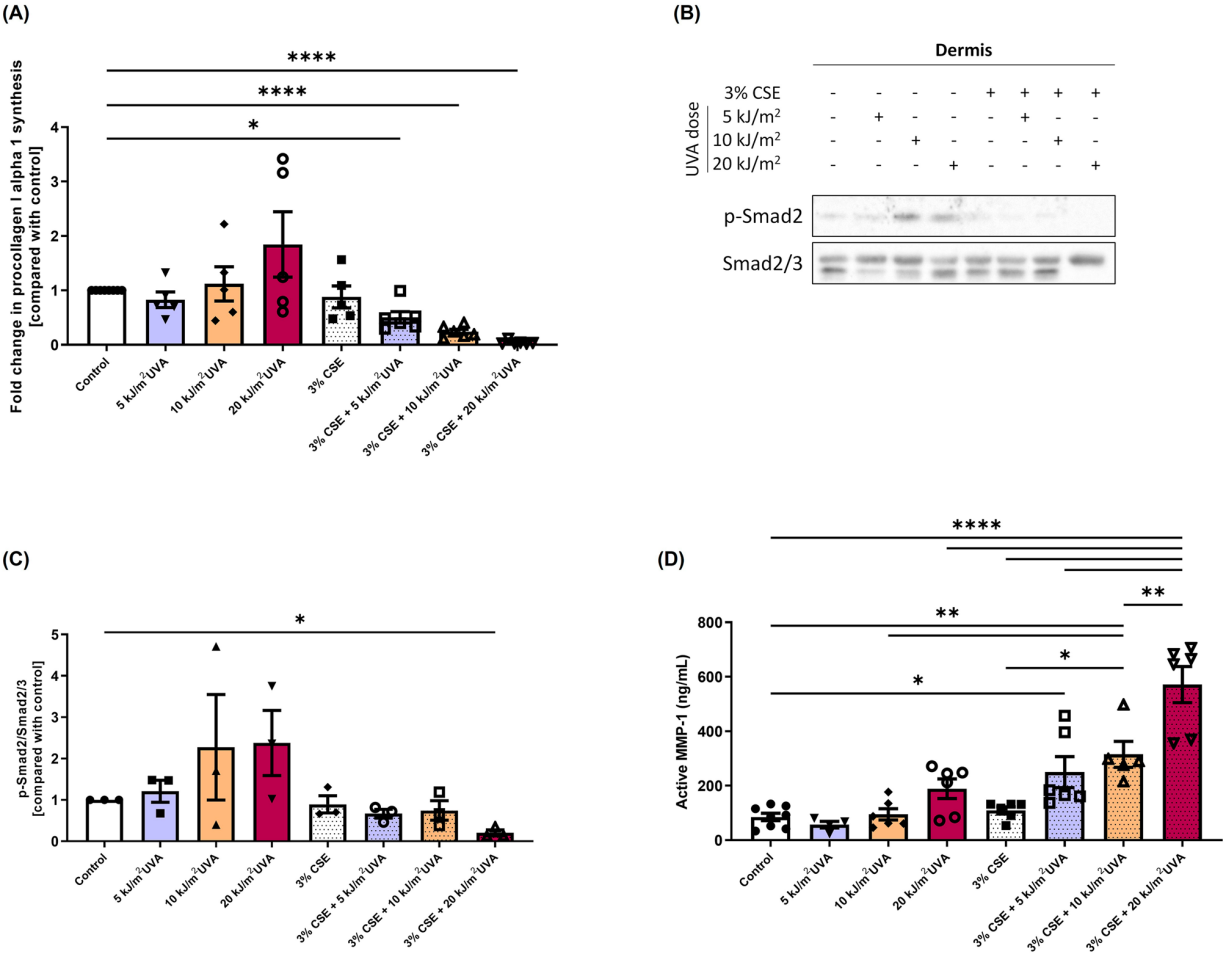
Effect of cigarette smoke extract (CSE) and SSL irradiation on procollagen synthesis via the inhibition of the TGF-β/Smad signaling pathway and matrix metalloproteinase (MMP) activity. (**A**) Procollagen I alpha 1 production assessed by ELISA in cell culture supernatants. Fold change is defined as the ratio of exposed substitutes’ procollagen quantity (in ng/mL) to the control (without treatment). (**B**) Protein expression of phosphorylated Smad2 in the dermis of reconstructed skin substitutes as determined by western blot. β-actin was used as the loading control. One representative blot is shown. (**C**) Densitometric quantification of the western blots. (**D**) MMP-1 activity in cell culture supernatants assessed by a fluorimetric assay (Human Active MMP-1 Fluorokine® E Kit). Analyses were performed with at least three different cell populations (3 ≤ N ≤ 5, 6 ≤ n ≤ 10). Data are presented as means of the different cell populations ± S.D. Statistical significance was determined using one-way ANOVA followed by Tukey’s post hoc test, * *p* value < 0.05, ** *p* value < 0.01, *** *p* value < 0.001, **** *p* value < 0.0001.

In order to study the contribution of chronic exposure to environmental factors individually or in combination to the skin aging exposome, healthy skin substitutes were exposed chronically to 3% CSE and/or SSL once per day during seven days. Their integrity was then analysed through histology (Fig. 3A). The co-exposure with 3% CSE and 20 kJ/m^2^ UVA, contrary to the other conditions, altered the epidermal layer by decreasing the thickness of the living epidermis (Fig. 3B; *p* value < 0.05 compared to control and 3% CSE alone), but it did not have any effect on the dermal thickness (Fig. 3C). Although no significant changes in the dermal thickness was observed in histology, the dermal layer alterations of the skin substitutes were investigated since the dermis, being a nonproliferating layer, is generally greatly affected by the extrinsic aging process. First, the synthesis of procollagen I was evaluated in the culture supernatant of skin substitutes. A significant decrease in procollagen I synthesis was observed in skin substitutes chronically exposed to 3% CSE combined with SSL exposure (5, 10 or 20 kJ/m^2^) of 0.5-fold (*p* value < 0.05), 0.2-fold (*p* value < 0.0001) and 0.05-fold (*p* value < 0.0001) respectively compared with the control without CSE and SSL irradiation (Fig. 4A). This decrease demonstrates the synergistic nature of this interaction, whereas SSL irradiation alone tended to increase the procollagen production (as seen with the 20 kJ/m^2^ UVA alone) and 3% CSE alone had no effect. Impairment of the transforming growth factor beta (TGF-β)/Smads signaling pathway has shown to be involved in the skin aging process in regard to the decrease in procollagen synthesis ^24–28^. To determine whether the TFG-β/Smads signaling pathway is involved in the synergy between cigarette smoke and solar radiation, the level of phosphorylation of Smad2 was assessed, together with total Smad2 levels, by western blot analyses of proteins extracted from the dermis of the skin substitutes (Fig. 4B and C). According to the immunostaining, a significant decrease in the ratio of phosphorylated-Smad2 to Smad2/3 was observed when 3% CSE was combined with 20 kJ/m^2^ UVA compared with the control (0.2-fold; *p* value < 0.05) while SSL irradiation alone seemed to increase the phosphorylation of Smad2 in certain samples and 3% CSE alone had no effect on the activation of this signal transducer, suggesting a role for the TGF-β/Smads signaling pathway in the inhibition of procollagen synthesis due to the synergy between the two extrinsic factors.

To evaluate the implication of matrix metalloproteinases (MMPs) in possible dermal alterations, MMP-1 activity was assessed in the culture supernatant of skin substitutes exposed or not to the studied factors (Fig. 4D). The activity of MMP-1 significantly increased when 3% CSE was combined with SSL irradiation (*p* value < 0.05 depending on the condition). The levels of active MMP-1 can be found in the Supplementary Table S2. The activity of MMP-1 was shown to be greater when the two extrinsic factors were combined than when they were used alone, confirming their synergistic effect. The level of tissue inhibitor of metalloproteinase (TIMP)-1 was also assessed, since it is generally produced concomitantly to attenuate the effect of MMP-1, and no significant increase was observed in TIMP-1 levels (Supplementary Fig. S1). These results demonstrate that the combination of two environmental factors can synergistically increase the activity of the proteases responsible for the degradation of key components of the dermis.

**Figure 5:**
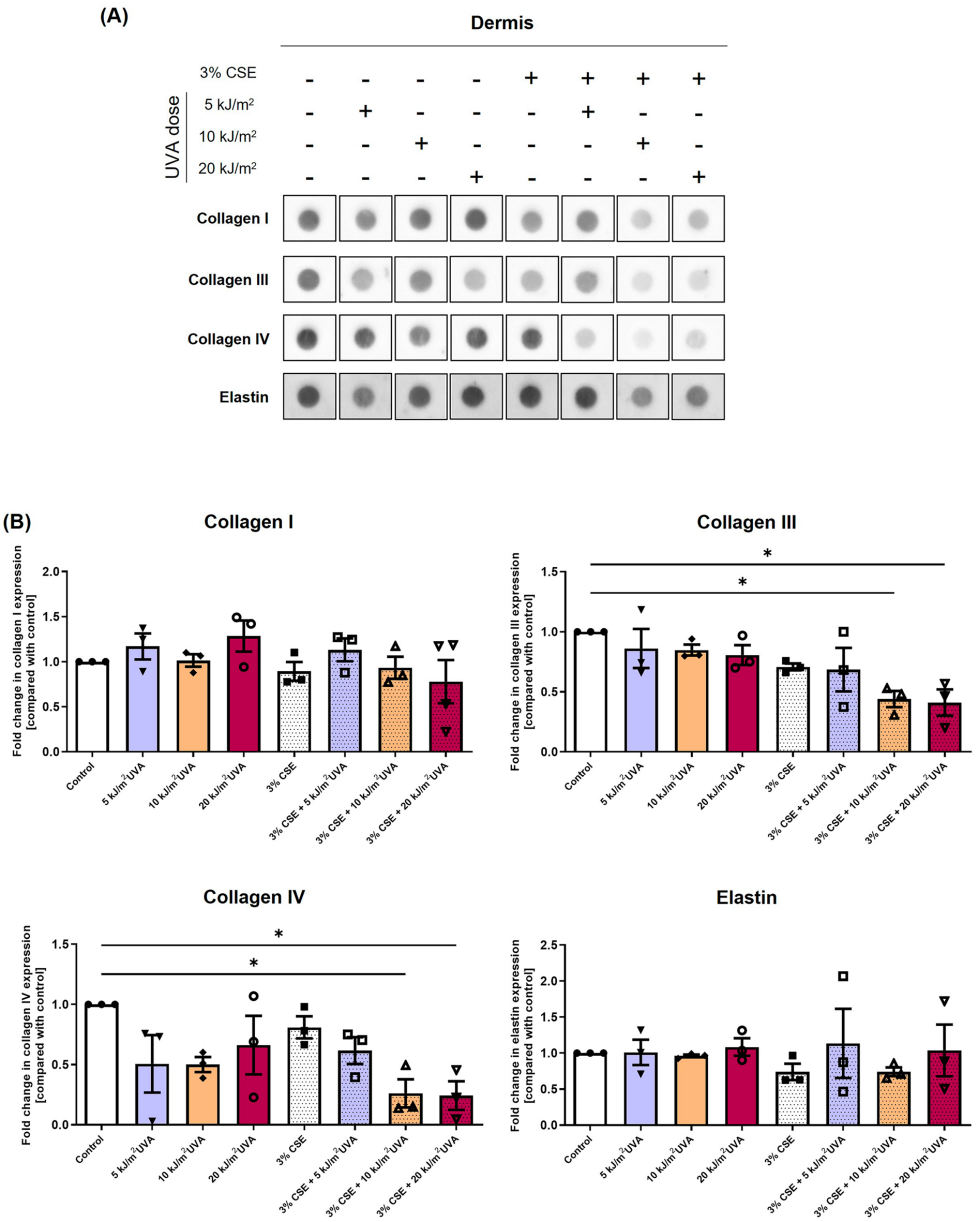
Effect of cigarette smoke extract (CSE) and SSL irradiation on dermal components of the extracellular matrix and basement membrane of reconstructed skin substitutes. (**A**) Dot blot analysis of collagen I, collagen III, collagen IV, and elastin. Dot blots come from the same membrane but were put in a more logical order, hence the black frame around each dot blot. (**B**) Densitometric quantification of the dot blots. GAPDH was used as loading control. Fold change is defined as the ratio of protein expression in exposed substitutes to that of the control (without treatment). Analyses were confirmed with three different cell populations (N = 3). Data are presented as means of the different cell populations ± S.D. Statistical significance was determined using one-way ANOVA followed by Tukey’s post hoc test, * *p* value < 0.05.

The increase in MMP activity suggests an increase in collagen degradation. Combined with the decrease in procollagen synthesis, this would lead to the decrease in collagen protein expression. The expression of collagen I and collagen III, the two most abundant types of collagens in the dermal layer, in addition to the expression of collagen IV, present in the basement membrane, and elastin, was investigated with indirect immunofluorescence staining and dot blot analysis. Immunofluorescence staining against the four markers did not allow any relevant conclusion to be drawn (Supplementary Fig. S2). Dot blot analyses of collagen I, III, and IV and elastin were then performed on proteins extracted from the dermis of skin substitutes (Fig. 5A). The results showed a significant decrease in collagen III and collagen IV expression when 3% CSE was combined with 10 and 20 kJ/m^2^ UVA, compared with the control (Fig. 5B). A 0.4-fold decrease in collagen III expression was observed for CSE + 10 kJ/m^2^ UVA and CSE + 20 kJ/m^2^ UVA compared with the control (*p* value < 0.05), while a 0.3-fold and 0.4-fold decrease in collagen IV was observed for CSE + 10 kJ/m^2^ UVA and CSE + 20 kJ/m^2^ UVA respectively compared with the control (*p* value < 0.05), confirming that the co-exposure provokes a synergistic effect, whereas their respective counterparts have no effect on the collagen protein expression. Taken together, these results showed that the synergy between cigarette smoke and solar rays accelerates the decrease in dermal component protein expression.

### The synergy activates MAPK signaling pathways

**Figure 6:**
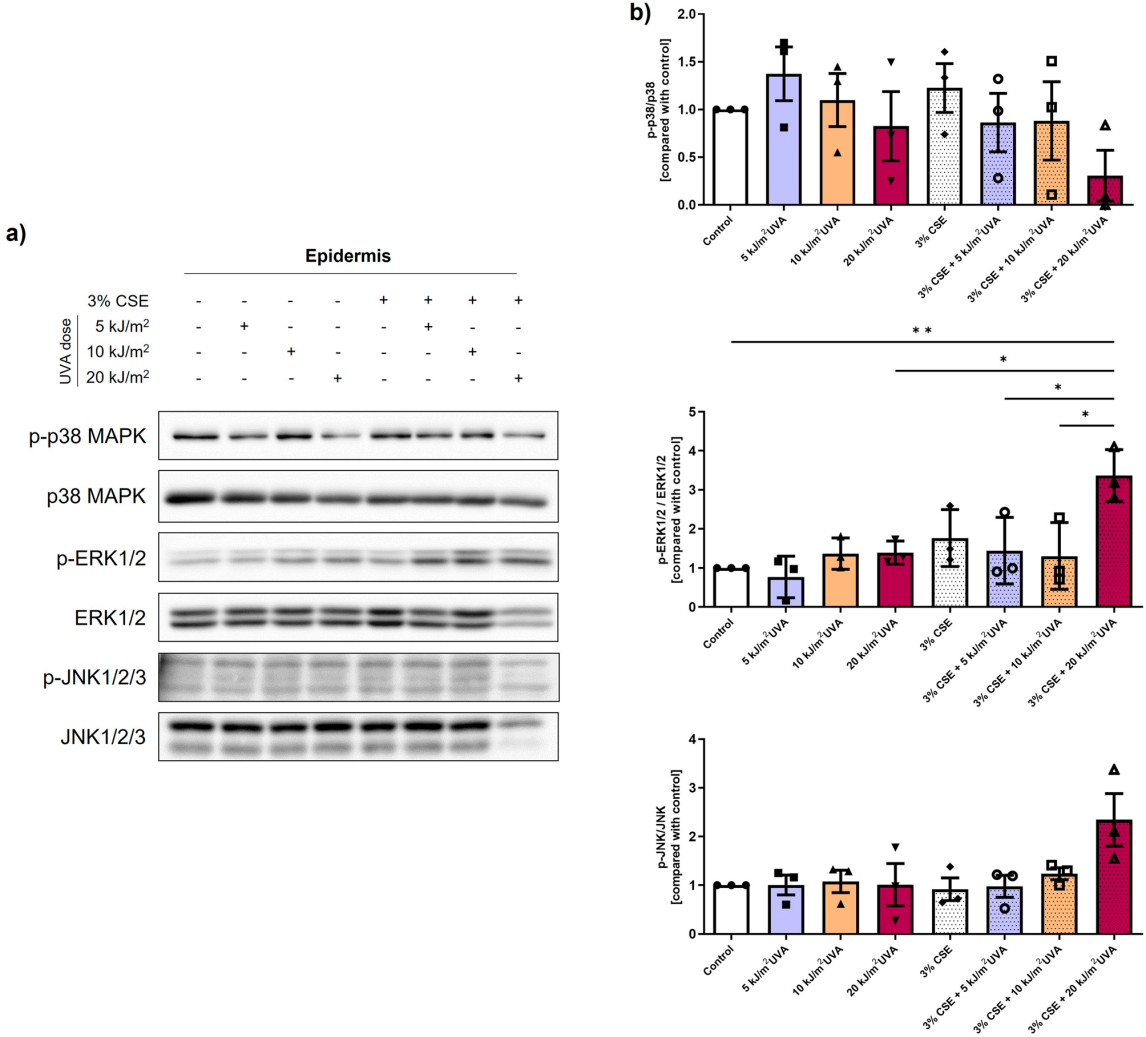
Activation of MAP kinase pathways by cigarette smoke extract (CSE) and SSL irradiation. (**a**) Levels of phosphorylated p38 MAPK, ERK1/2 and JNK in the epidermis of reconstructed skin substitutes as determined by western blot. β-actin was used as the loading control. One representative blot is shown. (**b**) Densitometric quantification of the western blots. Analyses were confirmed with three different cell populations (N = 3). Data are presented as means of the different cell populations ± S.D. Statistical significance was determined using one-way ANOVA followed by Tukey’s post hoc test, * *p* value < 0.05, ** *p* value < 0.01.

To determine if the activation of MAPK signaling pathways is involved in the synergy between CSE and SSL, the levels of total and phosphorylated p38 MAPK, ERK1/2 and JNK were analyzed by western blot on proteins extracted from the epidermis of the skin substitutes (Fig. 6). Solar radiation, especially UV, is known to induce the activation of the MAPK pathways^5,29^, which in turn can upregulate the MMP expression^30,31^ and alter the TGF-β/Smads pathway^29^. However, the activation of these pathways by cigarette smoke has not yet been investigated in the skin. The immunostaining showed a significant increase in the ratio of phosphorylated-ERK1/2 to ERK1/2 (3.4-fold) when 3% CSE was combined with 20 kJ/m^2^ UVA compared with the control (*p* value < 0.01), and it was also significant compared with the other combinations and its irradiation control (20 kJ/m^2^ UVA alone) (*p* value < 0.05), suggesting yet again the synergistic effect of the two extrinsic factors when combined. For the phosphorylation of JNK, a tendency with a 3.3-fold increase was observed with the 3% CSE + 20 kJ/m^2^ UVA when compared with the control (*p* value < 0.10). A slight, but not significant, decrease was observed in the ratio of phosphorylated-p38 MAPK/p38 MAPK for the 3% CSE + 20 kJ/m^2^ UVA condition. These results show the implication of the synergy between cigarette smoke and solar radiation in the regulation of ERK1/2 and JNK pathways.

### The combination between CSE and SSL increases the secretion of pro-inflammatory cytokines

To study the extent of the synergistic effect, the secretome of skin substitutes chronically exposed to cigarette smoke and solar rays was analysed. The levels of different cytokines were assessed by ELISA and by a cytokine array that detects the relative expression levels of 105 soluble cytokines (Supplementary Fig. S3). An increased expression of different pro-inflammatory cytokines, namely IL-6, G-CSF, GM-CSF, IL-1ra, IL-24 and CCL7, was observed with the environmental factors, alone or combined. The interpretation of these results and the discussion can be found in the Supplementary Material.

## Discussion

The “pro-aging” effect of solar radiation and cigarette smoke, independently, is well documented. The damage to the dermal connective tissue, including the reduced levels of the different types of collagens due to a decrease in their synthesis and an increase in their degradation via the MMPs, is the hallmark of extrinsic skin aging^32–37^. Despite this rather in-depth knowledge concerning single environmental factor exposure in the skin-aging phenomenon, very little is known about the effects that could result from a concomitant exposure to several environmental factors, nor about the mechanisms involved. The study of the interactions between factors is accompanied by its share of difficulties. Tissue-engineered skin substitutes are promising tools for the investigation of premature skin aging since they allow the study of different combinations and the contribution of each interaction. In the present study, the use of an in vitro reconstructed skin model allowed us to prove that cigarette smoke and solar rays can harm the skin and could accelerate the aging process. To our knowledge, this is the first study to report that cigarette smoke and solar rays synergistically increase the impairment of collagen protein expression, mediate the activation of MAPK signaling pathways and promote a pro-inflammatory environment.

In this study, a decrease in the amount of procollagen I alpha 1 was observed when CSE and SSL irradiation were combined (Fig. 4A). This effect was synergistic since their respective counterparts did not affected procollagen I synthesis. A proposed mechanism in skin aging for the downregulation in procollagen biosynthesis is the impairment of the TGF-β/Smads signaling pathway, since TGF-β is a cytokine that contributes primarily to this process^24–26^. Photoaged skin has been shown to express lower levels of TGF-β receptor II (TGFβRII) than normal skin^27,28^. This downregulation, which is upstream in the signaling pathway, provokes the inhibition of the whole signaling cascade by preventing Smad2 and Smad3 phosphorylation. Smad2/3 phosphorylation is dependent on the complex formation of a complex involving TGFβRII and TGFβRI. Indeed, the first step of in this signaling pathway is the binding of the ligand (TGF-β) to the TGFβRII, which will subsequently lead to the formation of the receptor complex with TGFβRI, as well as the phosphorylation of the latter^25^. Once the receptor complex is activated, it can in turn phosphorylate the receptor-regulated Smad proteins (R-Smads) Smad2 and Smad3, which will form heteromeric complexes with Smad4 that will translocate to the nucleus to regulate the gene transcription of procollagens^25^. Thus, the decrease in Smad2 activation observed in our study (Fig. 4B–C) suggests that TGFβRII could also be decreased in quantity by the synergy between cigarette smoke and sun rays, and that the downregulation of procollagen I alpha 1 is mediated by the TGF-β/Smads signaling pathway. However, the inhibition of Smad2 phosphorylation is probably not the only factor involved in the reduced synthesis of procollagen. Indeed, a significant decrease in Smad2 phosphorylation was only observed when CSE was combined with the highest dose of UVA (20 kJ/m^2^ UVA) and not with the other combinations, while a decrease in procollagen I levels was also induced when CSE was combined with 5 and 10 kJ/m^2^ UVA (Fig. 4A).

However, contrary to the decrease in procollagen I production when CSE was combined with SSL irradiation (3% CSE + 5 kJ/m^2^ UVA, 3% CSE + 10 kJ/m^2^ UVA and 3% CSE + 20 kJ/m^2^ UVA), an increased procollagen synthesis tendency was observed when skin substitutes were only irradiated with 20 kJ/m^2^ UVA (Fig. 4A). Several studies have shown that LED irradiation in the red spectrum or IR irradiation can lead to increase collagen biosynthesis^38–41^, which could explain this result. Indeed, a study from Barolet et al. (2009) showed that a 660 nm pulsed LED light can reverse the collagen downregulation and MMP-1 upregulation associated with skin aging^38^. Ayuk et al. and Fortuna et al. observed that red lasers at 660 and 670 nm respectively increased collagen levels^39,40^. Kim et al. observed that a single dose of IR can also increase the level of procollagen I without altering the expression of MMP-1. However, multiple IR doses reduced procollagen synthesis and increased MMP-1 expression^41^. The combination of red light (640 nm) and near-IR (830 nm) at low doses has also shown a beneficial effect for collagen synthesis^42^. This could thus explain the tendency of increased synthesis observed in the 20 kJ/m^2^ UVA control since the total solar spectrum, minus UVB and UVA2, was used in our study. However, it is difficult to compare our broad-spectrum SSL irradiation with these lasers, so to confirm, we could use a filter that blocks IR and re-evaluate the production of procollagen in another study. The opposite effect was observed, however, when 20 kJ/m^2^ UVA irradiation was combined with 3% CSE, suggesting that the harmful effect of the synergy outdoes the “beneficial” effect of red light and IR.

In addition to reduced collagen biosynthesis, an increased production of MMPs is another keystone of extrinsic aged skin and leads to irreversible consequences, more precisely the degradation of dermal components. MMPs are secreted by different cell types, such as fibroblasts, keratinocytes, and inflammatory cells^43,44^. In a co-culture of keratinocytes and fibroblasts, MMP-1 was found to be more abundantly secreted by keratinocytes than fibroblasts, while MMP-3 and TIMP-1 were produced mainly by fibroblasts^45^. An important observation in this study was that the quantities of all the mentioned MMPs were greatly increased in the presence of both keratinocytes and fibroblasts, showing the importance of the crosstalk between keratinocytes and fibroblasts in MMP production, and thus the relevance of a bilayer skin model. In our 3D skin model comprising keratinocytes and fibroblasts, we showed that MMP-1 activity was increased with the combination of cigarette smoke and solar rays regardless of the UVA dose compared with the different controls (Fig. 4D), resulting in a synergistical effect. Even though MMPs are responsible for the cleavage and degradation of collagens, MMP-1 is the only MMP that can cleave intact collagen fibers into triple helical domains^46^. It means that MMP-1 is the key protease that initiates the collagen degradation process, while the other MMPs can subsequently further degrade the cleaved collagen. The upregulation of MMP-1 activity that we observed in our study means that the degradation process of collagens must already have been initiated. The dot blot immunostaining results showed a decrease in collagen III levels when CSE was combined with 10 or 20 kJ/m^2^ UVA, while collagen I remained unaffected by such exposure (Fig. 5). Despite the fact that collagen I is the most abundant dermal collagen and that its quantity decreases with skin aging, collagen III is more sensitive to cleavage than collagen I, with a higher cleavage rate by nonspecific proteinases^47^. Moreover, its collagenase-sensitive region appears to unfold more easily than that of collagen I, which may explain the altered expression of collagen III assessed by dot blot and not of collagen I. However, the MMP-1 results, combined with the decrease in procollagen 1 synthesis shown in Fig. 4, suggest that a decrease in collagen I levels should occur. Thus, it suggests that this phenomenon might be more observable at later time points than the 24 h post-irradiation that we investigated in our study, since its cleavage rate is lower and its collagenase-sensitive region unfold less easily than that of collagen III.

The dermis is not the only structure of the skin affected by the downregulation of collagens. Collagen IV, a major component of the basement membrane produced by both keratinocytes and fibroblasts, is also reduced in quantity with skin aging^48,49^. We demonstrated that a decrease in collagen IV levels was indeed observed after the skin substitutes were chronically exposed to CSE and SSL irradiated (Fig. 5). Feru et al. (2016) showed that the synthesis of collagen IV is reduced in skin aging due to a decrease in the amounts of TGF-β1 and TGFβRII^48^. Therefore, the impairment of the TGF-β/Smads pathway observed in our study could also play a role in the decreased protein expression of collagen IV.

In order to investigate the mechanisms involved in the synergy, we studied the MAPK pathways and showed that both ERK1/2 and JNK are activated or tend to be activated by the concomitant exposure to cigarette smoke and solar rays (Fig. 6). These two MAPKs are known to be involved in MMP transcription by activating protein-1 (AP-1), a transcription factor predominantly resulting from the formation of a heterodimer between c-jun and c-Fos and mediating the regulation of several genes in response to various stimuli, including growth factors and cytokines^50^. The MAPK family, more specifically ERKs and JNKs, is in part responsible for the activation of the transcription factors and promoters involved in MMP transcription^30,31^. Indeed, ERKs and JNKs can phosphorylate, and thus activate, c-jun^51,52^. When c-jun dimerizes with c-Fos, it activates the transcription of several MMP promoters. It was shown in our study that the ERK and JNK pathways specifically were activated by the synergy between cigarette smoke and solar rays, when the CSE was combined with 20 kJ/m^2^ UVA. This suggests that the observed increase in MMP-1 activity (Fig. 4D) could be mediated in part by those MAPK pathways. It should still be noted that the MAPKs play a role in several other biological processes. For example, the ERK pathway also plays a role in the TGF-β/Smads signaling pathway. ERK is known to attenuate the accumulation of R-Smads in the nucleus and to increase the level of TG3-interacting factor (TGIF), a Smad co-repressor^29^.

**Figure 7:**
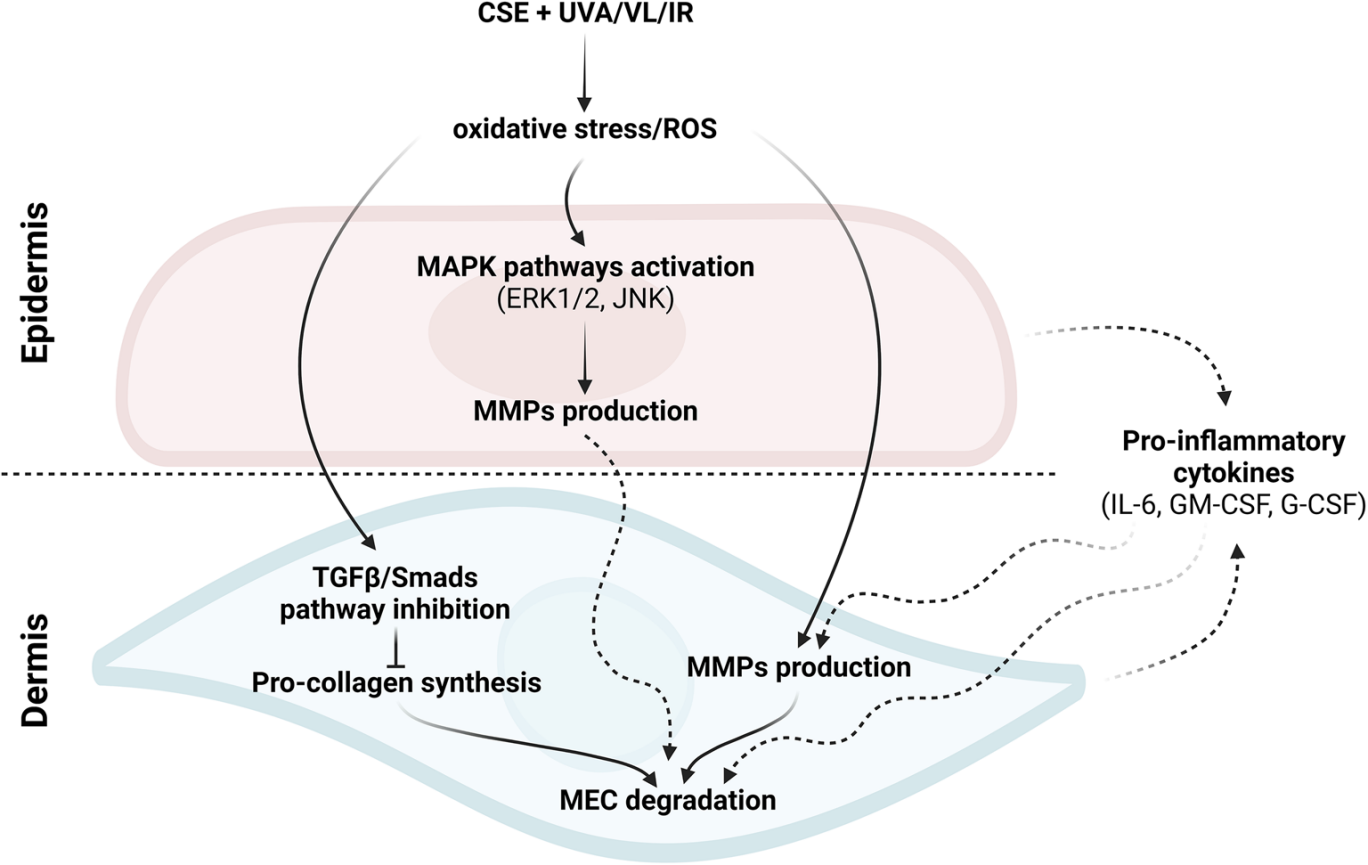
Proposed mechanisms of the synergistic effect between cigarette smoke extract (CSE) and SSL irradiation in skin aging.

Taken together, our results showed that the chronic exposition of 3D skin substitutes to cigarette smoke and solar rays, from UVA1 to IR, results in a synergy that could contributes to premature extrinsic skin aging. The skin substitutes exposed to both cigarette smoke and solar rays presented an aged profile with a decreased protein expression of collagens, induced by the inhibition of Smad2 phosphorylation leading notably to a decrease in procollagen synthesis, and the activation of MMP activity. The increased activity of the latter could be explained by the activation of MAPK pathways, more precisely ERK1/2 and JNK, with the co-exposure. Finally, an inflammaging environment could be observed with the increased quantities of several pro-inflammatory cytokines. Figure 7 summarizes a proposed mechanism for the synergistic effects following co-exposure with several of our hypotheses.

In our study, the environmental factors alone were not strong enough to cause an effect comparable to other studies performing acute exposures, since they usually use extreme doses. Hence the importance of our study, which demonstrates that even at physiological doses that seems harmless with a single environmental factor, the combination of the two factors provokes deleterious effects in the skin. Yet, it should be noted that the SSL irradiation doses, expressed in UVA, used in this study were based on chronic doses that can be received daily, i.e. between 5 and 20 min of sun exposure, and that the treatments and SSL irradiation were carried out for only 7 days. This means that the results observed would probably be more pronounced in reality, given that we are often exposed to the sun for longer periods of time and on a daily basis, not for only 7 days. Moreover, the CSE used in our study is an aqueous extract, meaning that only the soluble components of cigarette smoke are found in it. However, another study on water-soluble and hexane-soluble extracts showed that the latter significantly induce MMP-1 gene expression^36^, suggesting that the insoluble compounds of cigarette smoke could also induce deleterious effects. The results obtained in our study are therefore only an indication of the damage that may result from this harmful synergy. Even more worrying effects are to be expected with long-term exposure.

### Supplementary Information

Supplementary Information.

## Data Availability

No other dataset than what we have presented in the paper and supplementary materials in this study was generated or analyzed. All other information is available on request from the corresponding author.
