# Supplementary material for: The combination of cigarette smoke and solar rays causes effects similar to skin aging in a bilayer skin model
Source: Sci Rep. 2023 Oct 20;13:17969. doi: 10.1038/s41598-023-44868-z (PMC10589246; doi:10.1038/s41598-023-44868-z)
Supplement: Supplementary file 1 — Supplementary Information. [file 41598_2023_44868_MOESM1_ESM.docx]

**SUPPLEMENTARY MATERIALS AND METHODS**

**Indirect immunofluorescence staining**

Biopsies from the skin substitutes were embedded in Tissue-Tek O.C.T. Compound (Sakura Finetek, Torrance, CA, USA) and quick-frozen in liquid nitrogen to be then kept at -80°C until needed. 6 µm thick cryosections were cut on microscope slides and fixed in cold acetone for 10 min. The slides were incubated in the dark in a humidified chamber for 45 min in 1% BSA-PBS with the primary antibodies and for 30 min with the secondary antibodies under the same conditions. The primary and secondary antibodies used for the indirect immunofluorescence staining are listed in Supplementary Table S1. The slides were assembled with mounting media containing DAPI Fluoromount-G (SouthernBiotech, Birmingham, AL, USA), which stains the cell nucleus in blue, and observed using a Zeiss microscope with an AxioCam HR Rev3 camera (Carl Zeiss Meditec AG, Oberkochen, Germany).

**Table S1. Exhaustive information about the antibodies used for indirect immunofluorescence staining, and dot blot and western blot analyses.**

|  | Use | Name | Source | Supplier | Catalog number | Dilution |
| --- | --- | --- | --- | --- | --- | --- |
| Primary antibodies | Immunofluorescence and dot blot | **Anti-collagen I** | Rabbit | Cedarlane  (Burlington, ON, Canada) | CL50111AP-1 | IF: 1:200  DB: 1:2000 |
|  | Immunofluorescence and dot blot | **Anti-collagen III** | Rabbit | Cedarlane  (Burlington, ON, Canada) | CL50311AP | IF: 1:200  DB: 1:2000 |
|  | Immunofluorescence and dot blot | **Anti-collagen IV** | Rabbit | Abcam  (Cambridge, UK) | ab21295 | IF: 1:200  DB: 1:1000 |
|  | Immunofluorescence and dot blot | **Anti-elastin** | Rabbit | Abcam  (Cambridge, UK) | ab21610 | IF: 1:200  DB: 1:1000 |
|  | Western blot | **Anti-phospho-Smad2** | Rabbit | Cell Signaling  (Danvers, MA, USA) | 3108 | 1:500 |
|  | Western blot | **Anti-Smad2/3** | Rabbit | Millipore Sigma  (Oakville, ON, Canada) | 07-408 | 1:500 |
|  | Western blot | **Anti-phospho-p38 MAPK** | Rabbit | Cell Signaling  (Danvers, MA, USA) | 9211 | 1:1000 |
|  | Western blot | **Anti-p38 MAPK** | Rabbit | Cell Signaling  (Danvers, MA, USA) | 9212 | 1:10000 |
|  | Western blot | **Anti-phospho-p44/42 MAPK** | Rabbit | Cell Signaling  (Danvers, MA, USA) | 9101 | 1:1000 |
|  | Western blot | **Anti-p44/42 MAPK** | Rabbit | Cell Signaling  (Danvers, MA, USA) | 9102 | 1:1000 |
|  | Western blot | **Anti-phospho-JNK1/2/3** | Rabbit | ABclonal  (Woburn, MA, USA) | AP0276 | 1:500 |
|  | Western blot | **Anti-SAPK/JNK** | Rabbit | Cell Signaling  (Danvers, MA, USA) | 9252 | 1:1000 |
|  | Western blot | **Anti-β-actin** | Mouse | Sigma  (Oakville, ON, Canada) | A5441 | E: 1:30000  D: 1:1000 |
|  | Dot blot | **Anti-GAPDH** | Mouse | BioLegend  (San Diego, CA, USA) | 649202 | 1:8000 |
| Secondary antibodies | Immunofluorescence | **Anti-rabbit Alexa 488** | Donkey | Life Technologies  (Oakville, ON, Canada) | A21206 | 1:1600 |
|  | Dot blot and  western blot | **Anti-rabbit HRP labeled** | Goat | Jackson ImmunoResearch  (West Grove, PA, USA) | 111-035-003 | 1:60000 |
|  | Dot blot and  western blot | **Anti-mouse HRP labeled** | Goat | Jackson ImmunoResearch  (West Grove, PA, USA) | 115-035-003 | 1:60000 |

Abbreviations: IF: immunofluorescence, DB: dot blot, E: epidermis, D: dermis, HRP: horseradish peroxidase

**SUPPLEMENTARY RESULTS AND DISCUSSION**

**Impairment of the extracellular matrix of the dermis**

***MMP-1 increased activity***

The activity of MMP-1 was assessed in the culture supernatant of skin substitutes using a fluorescent assay and the mean value of active MMP-1 for each condition is presented in Supplementary Table S2.

**Table S2. Levels of active MMP-1 in the supernatant of skin substitutes exposed or not to the studied environmental factors.**

| Condition | Active MMP-1  (ng/mL) | |
| --- | --- | --- |
|  | **Mean** | **S.D** |
| Control | 85 | 39 |
| 5 kJ/m^2^ UVA | 57 | 25 |
| 10 kJ/m^2^ UVA | 95 | 51 |
| 20 kJ/m^2^ UVA | 189 | 88 |
| 3% CSE | 110 | 32 |
| 3% CSE + 5 kJ/m^2^ UVA | 250 | 138 |
| 3% CSE + 10 kJ/m^2^ UVA | 315 | 107 |
| 3% CSE + 20 kJ/m^2^ UVA | 571 | 162 |

***TIMP-1 synthesis***

The production of MMP-1 generally comes with the production of an MMP inhibitor, TIMP-1, that attenuates the effect of MMP-1. TIMP-1 levels were slightly increased (not significatively) with increasing UVA doses but were not higher with the concomitant exposure with CSE and SSL (Supplementary Figure S1), confirming that MMP-1 activity was not inhibited by TIMP-1. This slight increase in TIMP-1 with UVA doses probably means that a basal level of MMP-1 is inhibited by TIMP-1 following SSL irradiation, but that the level of activity of MMP-1 remains greater with the synergy. Thus, the synergy between the two factors does not promote the production of TIMP-1, an MMP-1 inhibitor, even though it promotes the production of the latter.

**
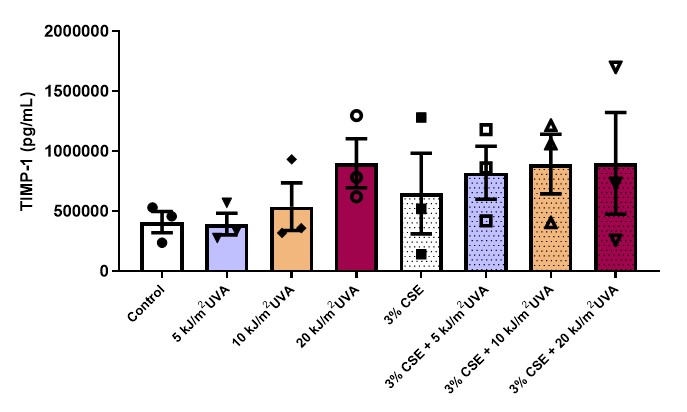
**

**Figure S1. Effect of cigarette smoke extract (CSE) and SSL irradiation on tissue inhibitor of metalloproteinase (TIMP)-1 expression.** TIMP-1 expression in cell culture supernatants. Analyses were confirmed with three different cell populations (N = 3, n = 6). Data are presented as means of the different cell populations ± S.D. Statistical significance was determined using one-way ANOVA followed by Tukey’s post hoc test.

***Indirect immunofluorescence staining against collagen I, III, IV, and elastin***

Indirect immunofluorescence staining against collagen I and collagen III showed a slight decrease in fluorescence intensity when SSL irradiated, but independently of the dose (whether it was 5, 10 or 20 kJ/m^2^ UVA) and whether it was combined or not with 3% CSE, compared with the control (Supplementary Figure S2, first and second columns respectively). A considerable decrease in the protein expression of collagen IV was observed when 3% CSE was combined with 10 or 20 kJ/m^2^ UVA (3% CSE + 10 kJ/m^2^ UVA and 3% CSE + 20 kJ/m^2^ UVA) compared with the control (Supplementary Figure S2, third column). As for elastin, in addition to its low fluorescence intensity in all conditions, no difference was observed between the conditions (Supplementary Figure S2, fourth column).

**
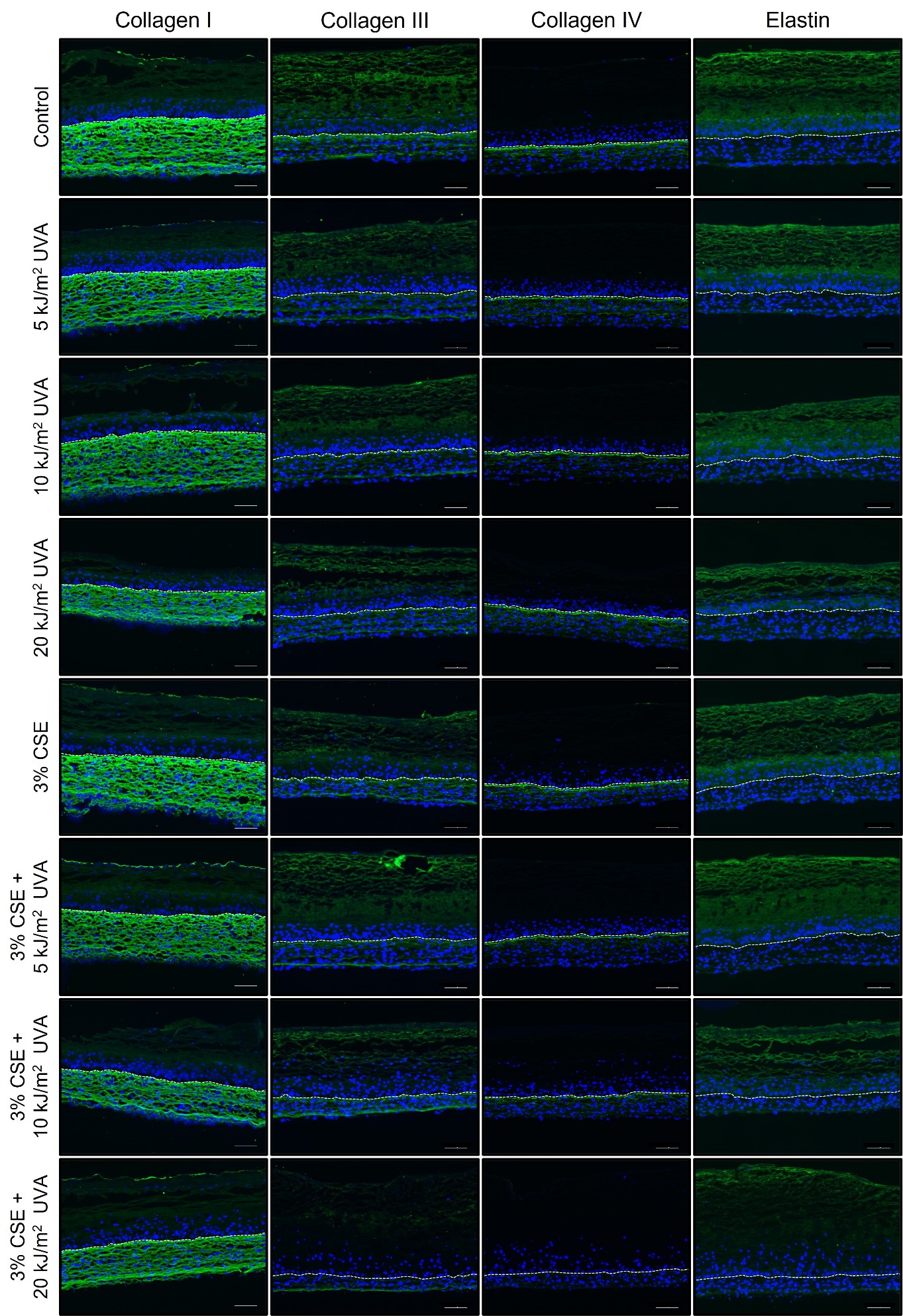
**

**Figure S2. Immunofluorescence staining of aging markers.** Collagen I (first column), collagen III (second column), collagen IV (third column) and elastin (fourth column) expression (green) in reconstructed skin substitutes not exposed (first panel), or chronically exposed to 5 kJ/m^2^ UVA (second panel), 10 kJ/m^2^ UVA (third panel), 20 kJ/m^2^ UVA (fourth panel), 3% CSE (fifth panel), 3% CSE and 5 kJ/m^2^ UVA (sixth panel), 3% CSE and 10 kJ/m^2^ UVA (seventh panel) or 3% CSE and 20 kJ/m^2^ UVA (eighth panel). The nuclei were stained with DAPI (blue). The dotted line represents the junction between the epidermis and the dermis. The part above the dotted line and beyond the blue nuclei is the stratum corneum (SC). Two skin substitutes for each condition were analyzed and confirmed with three different cell populations (N=3, n=6). Scale bar = 100 µm.

**Increased secretion of different pro-inflammatory cytokines**

IL-6, a known pro-inflammatory cytokine secreted after UV irradiation, was evaluated by ELISA in culture supernatant, and the results showed an increase in IL-6 levels after skin substitutes were chronically SSL irradiated or exposed to CSE (Supplementary Figure S3a). This increase was significant when both factors were combined, i.e. when skin substitutes were chronically exposed to 3% CSE and 10 or 20 kJ/m^2^ UVA, compared with the control. However, based on the slightly but not significant increased obtained for CSE exposition and SSL irradiation alone, this increase could result from an additive effect of both extrinsic factors, rather than a synergistic effect.

The cytokine array analysis performed on the culture supernatants of control, 10 kJ/m^2^ UVA, 3% CSE and 3% CSE + 10 kJ/m^2^ UVA conditions also produced interesting tendencies (Supplementary Figure S3b). According to the results, chronic exposure to 3% CSE, 10 kJ/m^2^ UVA and 3% CSE + 10 kJ/m^2^ UVA can modulate the secretion of several cytokines compared with the control (Supplementary Figure S3d). Among them, granulocyte colony-stimulating factor (G-CSF), granulocyte-macrophage colony-stimulating factor (GM-CSF), IL-1 receptor antagonist (IL-1ra), IL-24 and chemokine (C-C motif) ligand 7 (CCL7) tended to be upregulated in the three exposed conditions as compared with the control (Supplementary Figure S3c and d).

**
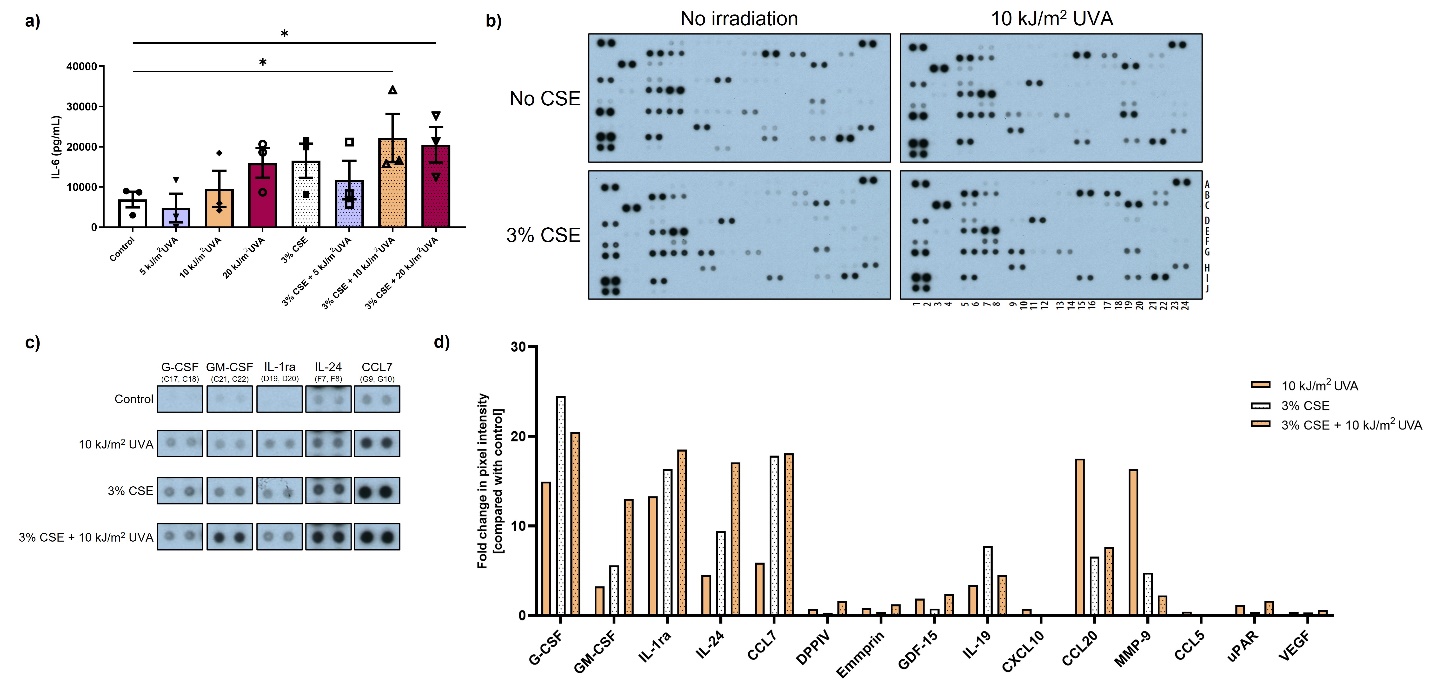
**

**Figure S3. Effect of cigarette smoke extract (CSE) and SSL irradiation on cytokines production in skin substitutes.** **a)** IL-6 secretion in cell culture supernatants. Analyses were confirmed with three different cell populations (N = 3, n = 6). Data are presented as means of the different cell populations ± S.D. Statistical significance was determined using one-way ANOVA followed by Tukey’s post hoc test, * *p*-value < 0.05. **b)** Comparison of detected cytokines secreted in cell culture supernatant between unexposed and nonirradiated skin substitutes (no CSE + no SSL irradiation; control), only irradiated at 10 kJ/m^2^ UVA (no CSE + 10 kJ/m^2^ UVA), only exposed to CSE (3% CSE + no SSL irradiation) and exposed to CSE and irradiated at 10 kJ/m^2^ UVA (3% CSE + 10 kJ/m^2^ UVA) using a Human XL Cytokine Array kit (R&D Systems). Information on each corresponding cytokine can be found in the manufacturer’s instructions. **c)** Altered cytokines in all three conditions (3% CSE, 10 kJ/m^2^ UVA and 3% CSE + 10 kJ/m^2^ UVA) compared with the control. **d)** Densitometric quantification of the dot blot duplicates from **b** of the cytokines altered the most. Fold change is defined as the ratio of exposed substitutes’ pixel intensity to the control (without treatment) pixel intensity. Analyses from the cytokine array were performed with one cell population and the supernatant of one skin substitute (N=1, n=1). Data are presented as means of the dot blot duplicates.

Along with IL-1 and TNF-α, IL-6 is a pro-inflammatory cytokine known to be part of inflammaging ^1^, but it could also indicate that skin cells have reached a senescent phenotype, since IL-6 is an important cytokine of the senescence-associated secretory phenotype (SASP) ^1-3^. However, other studies have suggested that IL-6 may also have an anti-inflammatory effect produced by suppressing the production of TNF-α and IL-1β and increasing the production of IL-1ra ^4,5^. In the present study, the quantity of IL-1ra, an antagonist of the IL-1 receptors and thus an antagonist of the IL-1 pathway, tended to increase in the culture supernatant of skin substitutes exposed chronically to the different factors, compared with the control. On the other hand, TNF-α and IL-1β were not detected with the cytokine array performed at day 21 of the air-liquid interface, which is in accordance with other studies by our team. Indeed, after 21 days at the air-liquid interface, cytokines are secreted in very low quantities compared with day 1 or day 7 ^6-9^. Thus, since IL-1β and TNF-α are present in very small amounts in our culture supernatant, it requires highly sensitive analyses in order to quantify them. Therefore, the absence of IL-1β and TNF‑α in this study does not necessarily mean that IL-6 acts as an anti-inflammatory mediator, and this phenomenon should be investigated further before any conclusions are drawn.

From the cytokine array performed on the culture supernatants of our skin substitutes, GM-CSF, IL-1ra and IL-24 were the most secreted in the 3% CSE + 10 kJ/m^2^ UVA condition. Interestingly, most of the cytokines that tended to be upregulated with exposure to the environmental factors (Supplementary Figure S3d) are cytokines involved in the recruitment of many immune cell types, such as monocytes, neutrophils, and macrophages, and in immune and inflammatory regulation, suggesting that an inflammatory environment is created by chronic exposure to extrinsic factors of skin aging.

Among these cytokines, an interesting cytokine that tended to be found in increased quantities in the supernatant of our skin substitutes chronically exposed to CSE and/or 10 kJ/m^2^ UVA is GM-CSF (Supplementary Figure S3c-d). GM-CSF is suspected to play a role in the formation of wrinkles. Indeed, the GM-CSF production induced by keratinocytes exposed notably to UVs stimulates fibroblasts, which will produce neprilysin (NEP) in return ^10,11^. NEP is responsible for the degradation of elastic fibers and thus impairs skin elasticity, leading to skin wrinkles ^10^. However, Mora Huertas et al. (2018) have shown that the elastase activity of NEP requires pre-damaged elastin fibers (e.g., by other proteases) in order to degrade them ^12^. The role of UVA in the induction of GM-CSF is still controversial. In Morisaki et al. (2010), UVB were shown to induce GM-CSF and IL-8 production in exposed keratinocytes but not in fibroblasts exposed to UVA, while Imokawa et al. (1996) found that IL-6, IL-8 and GM-CSF were produced in the culture supernatant of keratinocytes exposed to UVA ^11,13^. In our study, solar irradiation (from UVA1 to IR), CSE, and especially the combination of the two factors increased the production of GM-CSF by 3.2-fold, 5.6-fold, and 13-fold respectively. These results suggest that other wavelengths than UVB can stimulate the production of GM-CSF and that the combination of cigarette smoke and solar rays markedly induces the production of GM-CSF, although this experiment should be repeated. This induction by the synergy could lead downstream to the impairment of elastic fibers. GM-CSF production was known to be induced in cigarette-smoke-induced airway inflammation, but to the best of our knowledge, this is the first time that it has been reported in CSE-exposed skin ^14-17^.

Moreover, several of the cytokines observed in increased quantities in this study after exposition, namely IL-6, G-CSF and GM-CSF, are secreted in senescence-induced fibroblasts, suggesting that keratinocytes and/or fibroblasts in the presented model may have reached a senescent phenotype ^2,18^.

In summary, the smoke exposure and SSL irradiation of our skin substitutes enhanced the production of pro-inflammatory cytokines (Supplementary Figure S3), such as IL-6, GM-CSF, IL-1ra and IL-24, and thus revealed the presence of a pro-inflammatory environment, often mentioned in aging and called “inflammaging”. IL-6 is known to activate the ERK signaling pathway, but not JNK and p38 and has been shown to be involved in the modulation of MMP-1 production by dermal fibroblasts ^19,20^. Therefore, the increase in IL-6 levels observed in this study could indicate that the concomitant exposure provokes a pro-inflammatory environment and could be responsible, at least in part, for the activation of the ERK pathway and the increase in MMP-1 production.

**Original images of western blot and dot blot**

**Figure S4. Protein expression of a) p-Smad2 and b) Smad2/3 in the dermis of reconstructed skin substitutes as determined by western blot.**

**Figure S5. Protein expression of a) collagen I, b) collagen III, c) collagen IV and d) elastin in reconstructed skin substitutes as determined by dot blot.**

**Figure S6. Protein expression of a) p-p38 MAPK and b) p38 MAPK in the epidermis of reconstructed skin substitutes as determined by western blot.**

**Figure S7. Protein expression of a) p-ERK1/2 and b) ERK1/2 in the epidermis of reconstructed skin substitutes as determined by western blot.**

**Figure S8. Protein expression of a) p-JNK1/2/3 and b) JNK1/2/3 in the epidermis of reconstructed skin substitutes as determined by western blot.**

**SUPPLEMENTARY REFERENCES**

1 Pilkington, S. M., Bulfone-Paus, S., Griffiths, C. E. M. & Watson, R. E. B. Inflammaging and the Skin. *J. Invest. Dermatol.* **141**, 1087-1095, doi:10.1016/j.jid.2020.11.006 (2021).

2 Coppé, J.-P., Desprez, P.-Y., Krtolica, A. & Campisi, J. The Senescence-Associated Secretory Phenotype: The Dark Side of Tumor Suppression. *Annu. Rev. Pathol.* **5**, 99-118, doi:10.1146/annurev-pathol-121808-102144 (2010).

3 Ghosh, K. & Capell, B. C. The Senescence-Associated Secretory Phenotype: Critical Effector in Skin Cancer and Aging. *The Journal of investigative dermatology* **136**, 2133-2139, doi:10.1016/j.jid.2016.06.621 (2016).

4 Schindler, R. *et al.* Correlations and Interactions in the Production of Interleukin-6 (IL-6), IL-1, and Tumor Necrosis Factor (TNF) in Human Blood Mononuclear Cells: IL-6 Suppresses IL-1 and TNF. *Blood* **75**, 40-47, doi:10.1182/blood.V75.1.40.40 (1990).

5 Tilg, H., Trehu, E., Atkins, M., Dinarello, C. & Mier, J. Interleukin-6 (IL-6) as an anti-inflammatory cytokine: induction of circulating IL-1 receptor antagonist and soluble tumor necrosis factor receptor p55. *Blood* **83**, 113-118, doi:10.1182/blood.V83.1.113.113 (1994).

6 Lorthois, I., Simard, M., Morin, S. & Pouliot, R. Infiltration of T Cells into a Three-Dimensional Psoriatic Skin Model Mimics Pathological Key Features. *Int. J. Mol. Sci.* **20**, 1670, doi:10.3390/ijms20071670 (2019).

7 Morin, S., Simard, M., Flamand, N. & Pouliot, R. Biological action of docosahexaenoic acid in a 3D tissue-engineered psoriatic skin model: Focus on the PPAR signaling pathway. *Biochim. Biophys. Acta* **1866**, 159032, doi:10.1016/j.bbalip.2021.159032 (2021).

8 Morin, S., Simard, M., Rioux, G., Julien, P. & Pouliot, R. Alpha-Linolenic Acid Modulates T Cell Incorporation in a 3D Tissue-Engineered Psoriatic Skin Model. *Cells* **11**, 1513, doi:10.3390/cells11091513 (2022).

9 Rioux, G. *et al.* Development of a 3D psoriatic skin model optimized for infiltration of IL-17A producing T cells: Focus on the crosstalk between T cells and psoriatic keratinocytes. *Acta Biomater.* **136**, 210-222, doi:10.1016/j.actbio.2021.09.018 (2021).

10 Imokawa, G., Nakajima, H. & Ishida, K. Biological Mechanisms Underlying the Ultraviolet Radiation-Induced Formation of Skin Wrinkling and Sagging II: Over-Expression of Neprilysin Plays an Essential Role. *Int. J. Mol. Sci.* **16**, 7776-7795 (2015).

11 Morisaki, N. *et al.* Neprilysin Is Identical to Skin Fibroblast Elastase: ITS ROLE IN SKIN AGING AND UV RESPONSES. *J. Biol. Chem.* **285**, 39819-39827, doi:10.1074/jbc.M110.161547 (2010).

12 Mora Huertas, A. C. *et al.* Degradation of tropoelastin and skin elastin by neprilysin. *Biochimie* **146**, 73-78, doi:<https://doi.org/10.1016/j.biochi.2017.11.018> (2018).

13 Imokawa, G., Yada, Y., Kimura, M. & Morisaki, N. Granulocyte/macrophage colony-stimulating factor is an intrinsic keratinocyte-derived growth factor for human melanocytes in UVA-induced melanosis. *Biochem. J.* **313**, 625-631, doi:10.1042/bj3130625 (1996).

14 Dranoff, G. *et al.* Involvement of Granulocyte-Macrophage Colony-Stimulating Factor in Pulmonary Homeostasis. *Science* **264**, 713-716, doi:doi:10.1126/science.8171324 (1994).

15 Bozinovski, S., Jones, J. E., Vlahos, R., Hamilton, J. A. & Anderson, G. P. Granulocyte/Macrophage-Colony-stimulating Factor (GM-CSF) Regulates Lung Innate Immunity to Lipopolysaccharide through Akt/Erk Activation of NFkB and AP-1 in Vivo. *J. Biol. Chem.* **277**, 42808-42814, doi:10.1074/jbc.M207840200 (2002).

16 Bozinovski, S. *et al.* Innate immune responses to LPS in mouse lung are suppressed and reversed by neutralization of GM-CSF via repression of TLR-4. *American Journal of Physiology-Lung Cellular and Molecular Physiology* **286**, L877-L885, doi:10.1152/ajplung.00275.2003 (2004).

17 Shibata, Y. *et al.* GM-CSF Regulates Alveolar Macrophage Differentiation and Innate Immunity in the Lung through PU.1. *Immunity* **15**, 557-567, doi:10.1016/S1074-7613(01)00218-7 (2001).

18 Lim, H., Park, H. & Kim, H. P. Effects of flavonoids on senescence-associated secretory phenotype formation from bleomycin-induced senescence in BJ fibroblasts. *Biochem. Pharmacol.* **96**, 337-348, doi:<https://doi.org/10.1016/j.bcp.2015.06.013> (2015).

19 Firestein, G. S. & Manning, A. M. Signal transduction and transcription factors in rheumatic disease. *Arthritis Rheum.* **42**, 609-621, doi:<https://doi.org/10.1002/1529-0131(199904)42:4><609::AID-ANR3>3.0.CO;2-I (1999).

20 Dasu, M. R. K., Barrow, R. E., Spies, M. & Herndon, D. N. Matrix metalloproteinase expression in cytokine stimulated human dermal fibroblasts. *Burns* **29**, 527-531, doi:<https://doi.org/10.1016/S0305-4179(03)00154-2> (2003).
